# Itm2a Expression in the Developing Mouse First Lower Molar, and the Subcellular Localization of Itm2a in Mouse Dental Epithelial Cells

**DOI:** 10.1371/journal.pone.0103928

**Published:** 2014-07-31

**Authors:** Makiko Kihara, Tamotsu Kiyoshima, Kengo Nagata, Hiroko Wada, Hiroaki Fujiwara, Kana Hasegawa, Hirotaka Someya, Ichiro Takahashi, Hidetaka Sakai

**Affiliations:** 1 Laboratory of Oral Pathology, Division of Maxillofacial Diagnostic and Surgical Sciences, Faculty of Dental Science, Kyushu University, Fukuoka, Japan; 2 Section of Orthodontics and Dentofacial Orthopedics, Division of Oral Health, Growth and Development, Faculty of Dental Science, Kyushu University, Fukuoka, Japan; 3 Department of Endodontology and Operative Dentistry, Division of Oral Rehabilitation, Faculty of Dental Science, Kyushu University, Fukuoka, Japan; 4 Section of Implant and Rehabilitative Dentistry, Division of Oral Rehabilitation, Faculty of Dental Science, Kyushu University, Fukuoka, Japan; NIDCR/NIH, United States of America

## Abstract

Itm2a is a type II transmembrane protein with a BRICHOS domain. We investigated the temporospatial mRNA and protein expression patterns of Itm2a in the developing lower first molar, and examined the subcellular localization of Itm2a in murine dental epithelial (mDE6) cells. From the initiation to the bud stage, the *in situ* and protein signals of Itm2a were not detected in either the dental epithelial or mesenchymal cells surrounding the tooth bud. However, at the bell stage, these signals of Itm2a were primarily observed in the inner enamel epithelium of the enamel organ. After the initiation of the matrix formation, strong signals were detected in ameloblasts and odontoblasts. Itm2a showed a punctate pattern in the cytoplasm of the mDE6 cells. The perinuclear-localized Itm2a displayed a frequent overlap with the Golgi apparatus marker, GM130. A tiny amount of Itm2a was colocalized with lysosomes and endoplasmic reticulum. Minimal or no overlap between the Itm2a-EGFP signals with the other organelle markers for endoplasmic reticulum, lysosome and mitochondria used in this study noted in the cytoplasm. These findings suggest that Itm2a may play a role in cell differentiation during odontogenesis, rather than during the initiation of tooth germ formation, and may be related to the targeting of proteins associated with enamel and dentin matrices in the secretory pathway.

## Introduction

The development of mammalian organs is mediated through sequential and reciprocal epithelial-mesenchymal interactions. The development of mouse tooth germ, like many other organs, occurs by coordinated multi-step molecular interactions [1]. These molecular interactions are associated with more than 300 genes, and were related to the initiation and morphogenesis of the tooth germ [2]–[4]. However, the precise mechanisms underlying the molecular interactions related to tooth germ development are still unclear. Therefore, it is important to identify the regulatory genes that orchestrate these molecular interactions between dental epithelial and mesenchymal cells.

We previously performed cDNA subtraction between the mandibles on embryonic day 10.5 (E10.5) and E12.0 to investigate the regulatory genes associated with odontogenesis. Nine highly expressed positive clones from the E10.5 mandible and five highly expressed positive clones from the E12.0 mandible were obtained by colony array screening [5]. The expression of several of these genes is closely associated with the developing tooth germ [6]–[17]. Integral membrane protein 2a (Itm2a) was one of the highly expressed genes in the E12.0 mandible [5].

The Itm2a gene was originally isolated by a cDNA library subtraction of *in vitro* cultivated murine mandibular condyles [18]. The Itm2a protein and its related proteins, Itm2b and Itm2c, belong to the BRICHOS superfamily. These Itm2 proteins consist of four regions with distinct properties; the hydrophobic, linker, BRICHOS and C-terminal regions, as do other proteins in the BRICHOS superfamily [19], and are type II transmembrane proteins. Itm2a bears one BRICHOS domain at the C-terminal extracellular side [20], [21]. The expression of Itm2c is restricted to the brain [22], whereas Itm2b is ubiquitously expressed [23]. Itm2a is expressed in the developing muscle [24] and in chondrocytes of the resting zone of the murine growth plate [25]. Itm2a is also expressed in mature odontoblasts as well as T-cells and hair follicles [18], [26]. However, the function of Itm2a is so far unknown, except for its potential to regulate chondrogenic and myogenic differentiation [27]–[29]. In addition, only a few studies have so far addressed the intracellular localization of Itm2a protein. In the murine thymoma cell line, EL4, the Itm2a protein was observed in large cytoplasmic vesicles, and then translocated to the plasma membrane in the activated cells [26], whereas Itm2a protein was detected in the nuclei of human uterine endometrial stromal sarcoma cells [30]. Thus far, the function, expression pattern and intracellular localization of Itm2a mRNA and protein have not yet been fully elucidated.

In this study, the expression patterns of Itm2a mRNA and protein were examined in the course of tooth germ development from initiation to root formation. Furthermore, an immunocytochemistry study was performed using cultured dental epithelial mDE6 cells to confirm the intracellular localization of Itm2a protein in the dental epithelial cells. We herein address the possible functional roles of Itm2a during tooth development based on these results.

## Materials and Methods

### Animals

BALB/c embryos and postnatal mice were used in the study. The examined embryonic and postnatal stages were E10.5, E12, E13, E14, E15, E16, E17 and E18 after gestation and postnatal days 0 (PN0), PN1, PN2, PN3, PN4, PN5, PN10 and PN20. Adult BALB/c mice were obtained from Charles River Japan Inc. (Yokohama, Japan). All experimental procedures using mice were approved by the Animal Care and Use Review Committee, Kyushu University (Fukuoka, Japan) and performed according to the guidelines of the committee. After allowing 12 hr for the mating of 10–20 week-old-female and adult male BALB/c mice, the presence of successful insemination was determined based on the presence of a post-copulatory plug in the vagina, and this time point was defined as E0.5 [14], [16], [17].

### *In situ* hybridization of Itm2a mRNA

Antisense cRNA probes for a part of the Itm2a mRNA cording region (cds: 145–435) were designed according to the NCBI reference sequences (accession number: NM_008409.2). A template cRNA probe for Itm2a mRNA was prepared using PCR amplification with primer pairs, and was subcloned into the pGEM3Z plasmid (Promega, Madison, WI). These probes were labeled with digoxigenin (DIG)-UTP using the DIG RNA Labeling Kit (Roche, Mannheim, Germany). The gene-specific primers used for Itm2a were as follows: 5′-GGG AAT TCA TGG TGA AGA TCG CCT TCA AC-3′ and 5′-CCC AAG CTT AGG ATC CTC AGA ATC AAA GAA GC-3′.

The specificity and sensitivity of the probes were confirmed using membrane hybridization. *In situ* hybridization methods were carried out as described in previous studies [5]–[17] (Fig. S1). The embryos were fixed in 4% paraformaldehyde (PFA) in phosphate buffered saline (PBS)(pH 7.4) overnight at 4°C. PN3 to PN20 were demineralized with 5% ethylenediamine tetra-acetic acid (pH 7.4) at 4°C for three to five days depending on the stage. The samples were then embedded in OCT compound (Sakura Finetechnical Co. Ltd, Tokyo, Japan). Serial frontal cryosections were cut at 8-µm thickness, and were mounted on silane-coated glass slides (Muto pure chemicals, Tokyo, Japan). Itm2a sense probes were used as a control, and no hybridization signal was observed. “strong” and “weak” signals were used to describe for the relative evaluation of the signal intensity observed within the same tissue section. The developmental tooth germ process in the embryonic mandible was defined according to previous studies [8], [17].

### Immunohistochemistry

Serial frontal sections of the paraffin-embedded samples were cut at a thickness of 6-µm, and were mounted on silane-coated glass slides (Muto). The sections were de-waxed in xylene and re-hydrated in descending concentrations of ethanol. The slides were treated with antigen retrieval buffer (10 mM sodium citrate buffer, pH6.0) and microwaved for 5 min, and 10% goat serum (Nichirei Bioscience, Tokyo, Japan) was used to block non-specific immunoreactions. The specimens were incubated with the primary antibody, rabbit polyclonal anti-Itm2a antibody (1∶1000, 18306-1-AP; Proteintech Group, Inc., Chicago, IL) at 4°C. The primary antibodies against Itm2a were visualized using an Alexa Fluor 568-conjugated anti-rabbit secondary antibody (1∶2000, A11011; Invitrogen, Carlsbad, CA). Finally, the immunostained sections were counterstained with 4′6-diamidino-2-phenylindole (DAPI, Dojindo, Kumamoto, Japan). The sections were rinsed in PBS three times at every interval between the steps.

The immunoreactivity of hepatocytes (Fig. S2) and chondrocytes was used as a positive control to develop the immunohistochemical staining procedures based on the manufacturer’s instructions and previous studies [18], [20], [31].

### Cell line and cell culture

The mDE6 cells, a mouse dental epithelial cell line [16], [17], were maintained in D-MEM/F-12 (Invitrogen) supplemented with 10% fetal bovine serum (FBS)(Invitrogen), 100 U/ml penicillin and 100 µg/ml streptomycin (Invitrogen) in a humidified atmosphere of 5% CO_2_ at 37°C.

### Gene transfection and immunocytochemistry with confocal microscopy

The coding region of the murine Itm2a cDNA (cds 145–933) was prepared using PCR amplification with primer pairs and was inserted into the pEGFP-N1 vector (Clontech, CA). The gene-specific primers used for Itm2a were as follows: 5′-CGG AAT TCA TGG TGA AGA TCG CCT TCA AC-3′ and 5′-GGG GTA CCG TCT CCT GAC AGA TCT TGG TTT C-3′. After confirming the base sequences of the inserted Itm2a cDNA, the pEGFP-N1 encompassing Itm2a cDNA were digested with Hind III and Not I. Subsequently, the Itm2a cDNA fused with EGFP cDNA was transferred into pcDNA3.1(+)/Hygro (Invitrogen). The mDE6 cells were stably transfected with pcDNA3.1(+)/Hygro encompassing Itm2a-EGFP cDNA using Lipofectamine LTX (Invitrogen) according to the manufacturer’s instructions. These transfectants were isolated after selection with 400 µl/ml hygromycin (Invitrogen) for three weeks. Thereafter, these mDE6 cells with stable Itm2a-EGFP expression were called Itm2a-EGFP-mDE6 cells. Some cells were transfected with pEGFP-N1 alone and were then used as a control (Fig. S3).

For the immunocytochemistry study to observe the subcellular localization of the Itm2a protein, the Itm2a-EGFP-mDE6 cells were seeded onto cover glass chambers (Thermo, Yokohama, Japan; 5.0×10^4^ cells/cm^2^) and were cultured in D-MEM/F-12 supplemented with 10% FBS. At 24 hr after seeding, the transfected cells were fixed in 4% PFA in PBS for 10 min. Some fixed cells were permeabilized with cold methanol for 10 min, followed by reaction with an antibody against endoplasmic reticulum (ER). The others were permeabilized with 0.5% Triton-X-100 (Wako, Osaka, Japan) for five minutes before incubation with the other antibodies. The cells were treated with 10% goat serum (Nichirei) to prevent non-specific binding for 20 min. The cells were then incubated with an antibody against the ER (1∶200, #2446, PDI antibody; Cell Signaling Technology, Tokyo, Japan), Golgi apparatus (1∶1000, G7295, Anti-GM130, C-terminal; Sigma-Aldrich, Tokyo, Japan), lysosome (1∶200, L0668, Anti-LAMP2; Sigma-Aldrich) or mitochondria (1∶200, M6319, Anti-Mitofusion-2, N-terminal; Sigma-Aldrich) at room temperature for 1 hr. Antibodies were visualized using an Alexa Fluor 568-conjugated anti-rabbit antibody (1∶2000, A11011; Invitrogen) at room temperature for 30 min. Cell nuclei were counterstained with DAPI for five minutes. The EGFP-fusion proteins expressed in the Itm2a-EGFP-mDE6 cells were confirmed using an antibody against Itm2a (Fig. S4). Images were obtained with a Nikon C2 confocal laser scanning microscope (Nikon Instech, Tokyo, Japan) and a CCD CoolSNAP HQ camera (Roper Scientific, Inc., Arizona) driven by the NIS-Elements software program (Nikon Instech).

## Results

*In situ* hybridization and the immunohistochemical analyses were performed to investigate the temporal and spatial expression of the Itm2a mRNA and protein during the mouse molar development from E10.5 to PN5. The developmental tooth germ stages in the embryonic mandible were defined according to our previous studies [8], [14], [17].

### Expression pattern of Itm2a mRNA during mouse molar development

#### Initiation (E10.5) and thickening of the dental epithelium (E12)

On E10.5, the oral mucosal epithelium to form the tooth bud was not apparently thickened. On E12, thickening of the oral mucosal epithelium was locally observed, thus indicating the presence of tooth bud formation.

**Figure 1:**
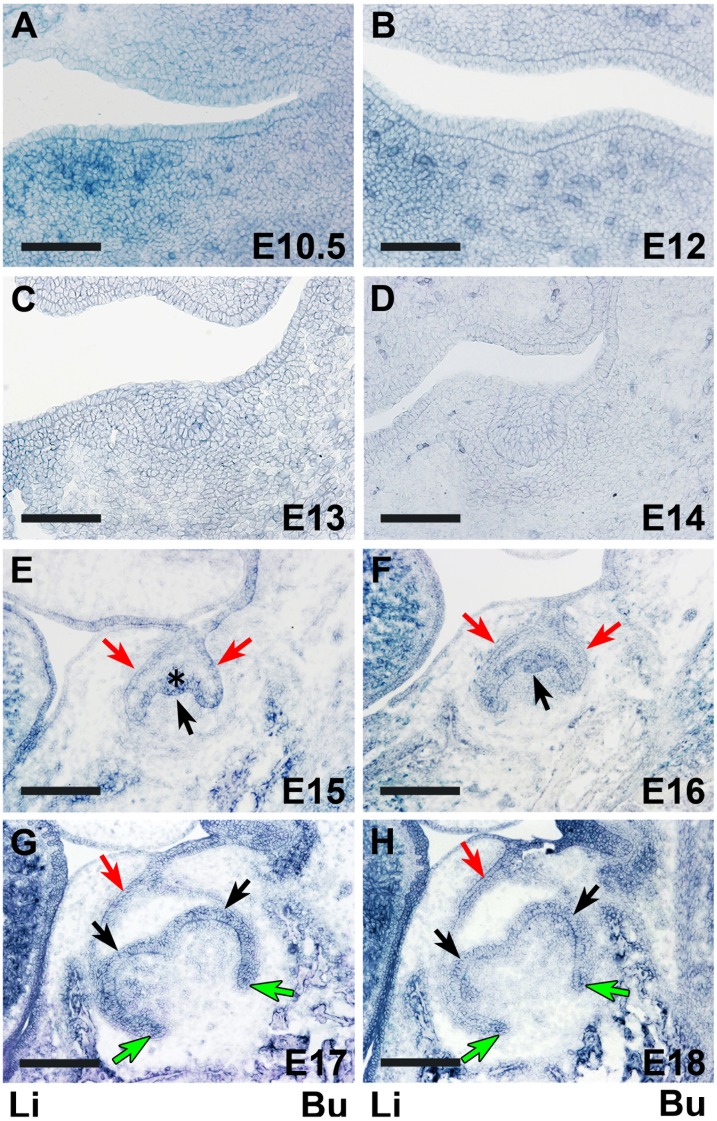
The *in situ* expression of Itm2a mRNA in the tooth germ during the fetal stages. The *in situ* Itm2a expression was temporally and spatially stage-specific during the tooth germ development. **A & B.** The *in situ* Itm2a signals were not detected in either the epithelial or ectomesenchymal cells corresponding to the predicted lower first molar region before or after oral epithelium thickening. **C & D.** At the bud stage, the *in situ* expression of Itm2a was not observed in the tooth germ. **E & F.** At the cap stage, the *in situ* expression of Itm2a was observed in outer enamel epithelial cells (red arrows), inner enamel epithelial cells (black arrows) and in the enamel knot (asterisk). **G & H.** At the early bell stage, the *in situ* expression of Itm2a was found in inner enamel epithelial cells (black arrows), epithelial cells in the tip of the cervical loop (green arrows) and in the outer enamel epithelial cells (red arrows). Li; lingual side, Bu; buccal side. Scale bars: 100 µm (A–D), 200 µm (E–H).

On these embryonic days, an *in situ* signal for Itm2a mRNA was detected in the ectomesenchyme of the mandible, whereas no specific signal for Itm2a mRNA was found in the epithelial and mesenchymal cells corresponding to the predicted lower first molar region (Fig. 1A, B). Instead, the *in situ* signal for Itm2a mRNA, especially on E12, was detected in the central area of the mandible, which corresponded to a part of the tongue primordium (data not shown). This result was in agreement with our findings in a previous study [5].

#### Bud stage (E13–E14)

The epithelial cells invaginated into the ectomesenchyme of the mandible to form the tooth bud. The *in* ****** *situ* signal of Itm2a mRNA was not yet observed in the epithelial cells of the tooth bud and in the mesenchymal cells surrounding the invaginated epithelial tooth bud (Fig. 1C, D).

#### Cap stage (E15–E16)

At this stage, the tooth bud grew larger, took on the appearance of a cap, and became an enamel organ. On E15, the *in* ****** *situ* signal of Itm2a mRNA was observed in the outer enamel epithelium, inner enamel epithelium and central epithelial cells of the enamel organ. A strong signal was detected in the primary enamel knot (Fig. 1E). A weak *in* ****** *situ* signal was observed in the dental lamina. A very weak *in* ****** *situ* signal was also observed in the dental papilla and dental sac. Some signals were noted in the blood vessels in the dental pulp.

The expression patterns of Itm2a in the dental epithelial and mesenchymal cells on E16 (Fig. 1F) were almost identical to those on E15. Itm2a expression was detected in the dental sac surrounding the enamel organ.

#### Early bell stage (E17–E18)

The enamel organ came to resemble a bell during this stage. The periphery of the enamel organ extended to the mesenchymal tissue and enfolded the dental papilla. On E17, the *in* ****** *situ* signal of Itm2a mRNA was detected in the inner enamel epithelium, and the intensity appeared to be slightly increased (Fig. 1G). The *in* ****** *situ* signal of Itm2a mRNA was also observed in the outer enamel epithelium and central epithelial cells of the enamel organ near the cervical loop. The *in* ****** *situ* Itm2a signal in the inner enamel epithelium was stronger than that observed in the outer enamel epithelium. A weak *in* ****** *situ* signal was observed in the dental papilla, and in the dental sac surrounding the enamel organ.

The expression pattern of Itm2a mRNA in the dental epithelial cells on E18 (Fig. 1H) was similar to that on E17. The *in situ* Itm2a expression was detected in the dental papilla localized at the site where the cusps had formed (Fig. 1H). The *in situ* signal was still weak in the dental sac surrounding the enamel organ.

#### Late bell stage (PN0–PN4)

**Figure 2:**
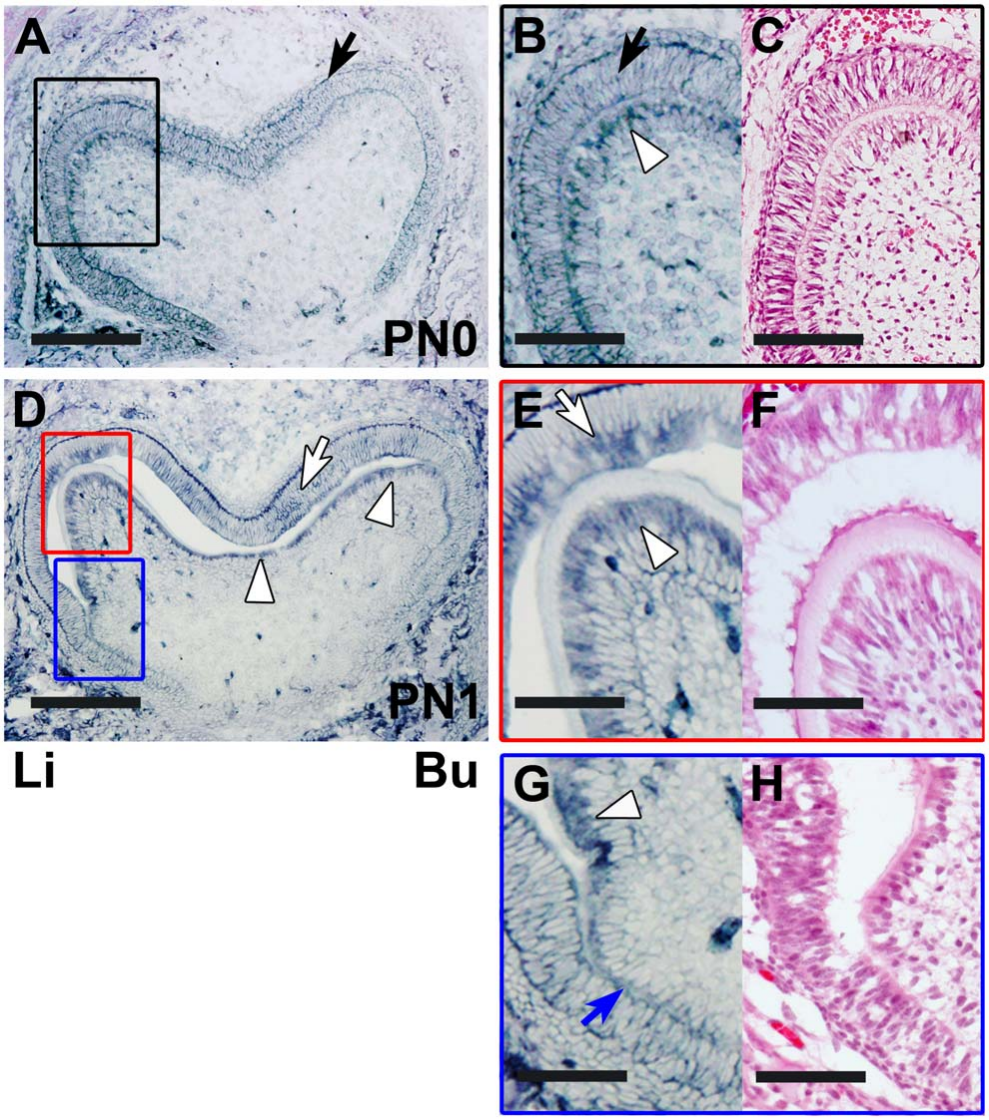
The *in situ* expression of Itm2a mRNA in the developing tooth germ at the late bell stage. **A.** On PN0, the *in situ* expression of Itm2a was detected in the inner enamel epithelial cells (black arrow). **B.** The boxed area in A is shown at a higher magnification. The *in situ* expression of Itm2a was detected in the inner enamel epithelial cells (black arrow) and odontoblasts (white arrowhead). **C.** An HE-stained section of the tooth germ on PN1 is shown, corresponding to the region shown in B. **D.** On PN1, the initiation stage of matrix formation, *in situ* Itm2a signals were observed in the ameloblasts (white arrow) and odontoblasts (white arrowheads). **E & G.** The red- and blue-boxed areas in D are shown at a higher magnification, respectively. The *in situ* expression of Itm2a was detected in the ameloblasts (white arrow) and odontoblasts (white arrowheads). An *in situ* Itm2a signal was also observed in preameloblasts (blue arrow). **F & H.** HE-stained sections of the tooth germ on PN1 are shown, corresponding to the regions shown in E and G, respectively. Li; lingual side, Bu; buccal side. Scale bars; 200 µm (A, D), 100 µm (B, C, E–H).

**Figure 3:**
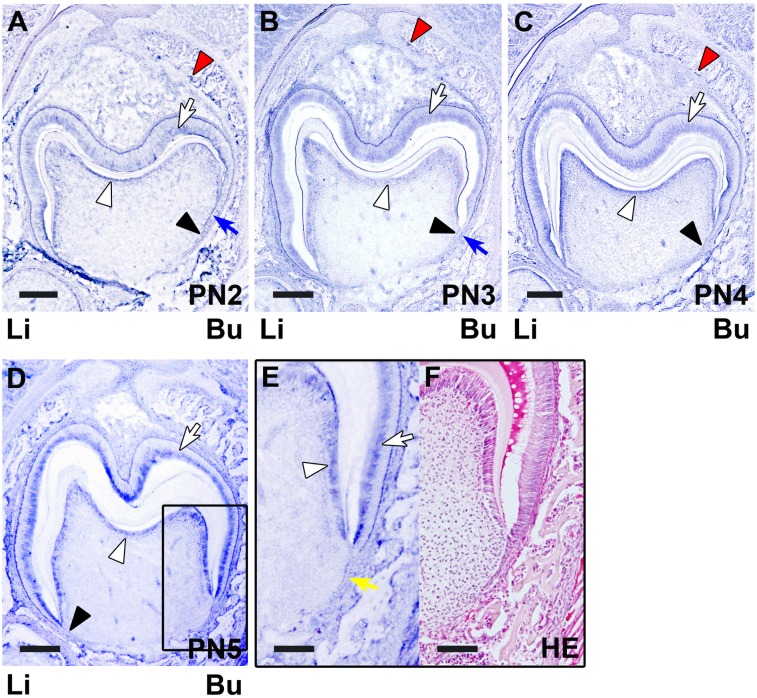
The *in situ* expression of Itm2a mRNA in the developing tooth germ at the matrix formation stage and the initiation stage of root formation. **A–C.** During matrix formation, an *in situ* Itm2a signal was detected in the ameloblasts (white arrows), preameloblasts (blue arrows), odontoblasts (white arrowheads) and preodontoblasts (black arrowheads). The signal was also detected in the osteoblasts (red arrowheads) of the bone tissue surrounding the tooth germ. **D.** The *in situ* Itm2a signal was observed in the ameloblasts (white arrow), odontoblasts (white arrowhead) and preodontoblasts (black arrowhead). **E.** The boxed area in D is shown at a higher magnification. The *in situ* expression of Itm2a was detected in the ameloblasts (white arrow) and odontoblasts (white arrowhead). Itm2a expression was also detected in the HERS (yellow arrow). **F.** An HE-stained section of the tooth germ on PN5 is shown. Li; lingual side, Bu; buccal side. Scale bars; 200 µm (A–D), 100 µm (E, F).

At the late bell stage, the inner enamel epithelium became ameloblastic, and the dental pulp cells facing the inner enamel epithelium differentiated into odontoblasts (Fig. 2). Simultaneously, ameloblasts and odontoblasts secreted enamel and dentin matrices, respectively (Figs. 2D**–**H and 3).

On PN0, when neither enamel nor dentin matrices had been formed, Itm2a mRNA was expressed throughout the preodontoblasts localized at the presumptive cusp sites of the dental pulp and the inner enamel epithelium (Fig. 2A). The outer enamel epithelium and the stratum intermedium also exhibited weak positive signals. The expression of Itm2a mRNA was observed in the preodontoblasts localized at the presumptive cusp sites of the dental pulp (Fig. 2A–C). *In situ* Itm2a expression was also detected in the blood vessels in the dental pulp.

On PN1, the formation of both enamel and dentin matrices between ameloblasts and odontoblasts was seen at the presumptive cusp site in the tooth germ. Strong *in situ* Itm2a expression was detected in ameloblasts and odontoblasts (Fig. 2D). *In situ* Itm2a expression was detected in the preameloblasts, which were localized at the central groove sites near the cervical loop (Fig. 2E–H), and in the stratum intermedium. The weak *in situ* Itm2a expression was also found in the preodontoblasts. The *in situ* Itm2a signal intensity was increased in the dental sac.

During crown matrix formation (PN2–PN4), strong *in situ* Itm2a expression was detected in the ameloblasts and odontoblasts (Fig. 3A–C). Strong *in situ* Itm2a expression was maintained in the ameloblasts and odontoblasts. On PN2–3, *in situ* Itm2a expression was also observed in the preameloblasts and preodontoblasts. On PN4, *in situ* expression was detected in the preodontoblasts. In addition, strong *in situ* Itm2a expression was observed in the osteoblasts which rimmed the alveolar bone surrounding the tooth crown.

#### Tooth root formation stage (PN5 and PN10)

Enamel matrix formation was terminated by this stage, and root formation was initiated. The epithelial cells located at the cervical loop of the enamel organ in the developing tooth germ proliferated and extended toward the mesenchymal tissue, thus forming Hertwig’s epithelial root sheath (HERS). Tooth root formation was also observed on PN10.

**Figure 4:**
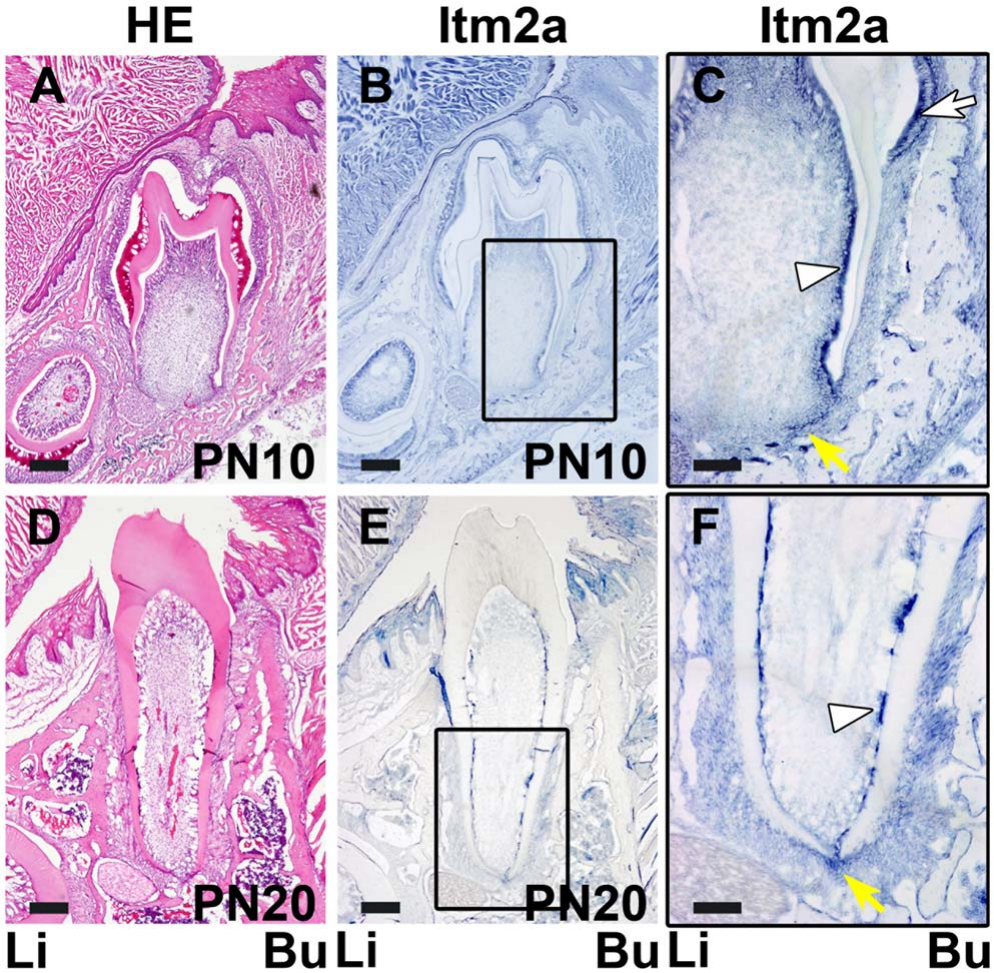
The *in situ* expression of Itm2a mRNA in the developing tooth germ at the tooth root formation and the tooth eruption stages. **A.** An HE-stained section shows the tooth germ on PN10 at the tooth root formation stage. **B.** The *in situ* signal of Itm2a was observed in the tooth germ on PN10. **C.** The boxed area in B is shown at a higher magnification. The *in situ* expression of Itm2a was detected in the ameloblasts (white arrow), odontoblasts (white arrowhead) and HERS (yellow arrow). **D.** An HE-stained section shows the tooth germ on PN20 at the tooth eruption stage. On PN20, the root formation is almost completed, and the crown is exposed. **E.** The *Itm2a* expression was observed in the odontoblasts and HERS. **F.** The boxed area in E is shown at a higher magnification. The *in situ* expression of Itm2a signal was detected in the odontoblasts (white arrowhead) and HERS around the root apex (yellow arrow). Li; lingual side, Bu; buccal side. Scale bars; 200 µm (A, B, D, E), 100 µm (C, F).

A strong *in situ* signal for Itm2a mRNA was observed in the ameloblasts and odontoblasts (Figs. 3D–F and 4A–C), but the signal intensity became reduced in the odontoblasts and ameloblasts in the presumptive cusp sites on PN5. The HERS also exhibited a strong *in situ* signal for Itm2a mRNA (Fig. 3E, F and Fig. 4B, C). The outer enamel epithelium and the stratum intermedium exhibited only very weak positive signals on PN5 and PN10.

#### Tooth eruption stage (PN20)

Tooth root formation was almost completed by this stage, and the tooth crown was exposed at the surface of oral mucosa (Fig. 4D**–**F). A strong *in* ****** *situ* signal for Itm2a mRNA was observed in the odontoblasts and HERS around the root apex (Fig. 4E, F). The developing periodontal membrane exhibited weak positive signals.

### Expression pattern of the Itm2a protein during mouse Molar development

**Figure 5:**
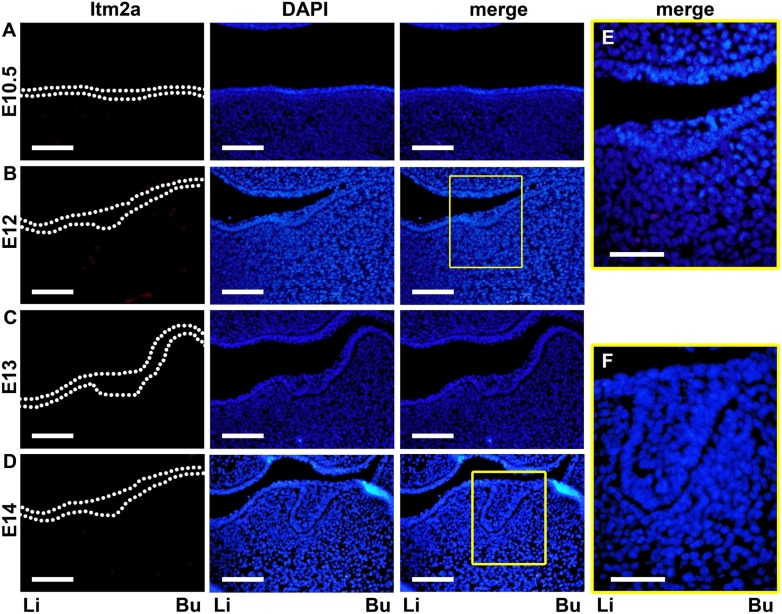
The Itm2a protein expression in the tooth germ on E10.5–E14. **A–D.** The expression of the Itm2a protein was not observed in the epithelial or ectomesenchymal cells corresponding to the predicted lower first molar region either before or after oral epithelium thickening, or at the bud stage. The dotted lines indicate the contours of the oral epithelial layer and/or tooth bud. **E & F.** The boxed areas in B and D are respectively shown at a higher magnification. Li; lingual side, Bu; buccal side. Scale bars; 100 µm (A–D), 50 µm (E, F).

**Figure 6:**
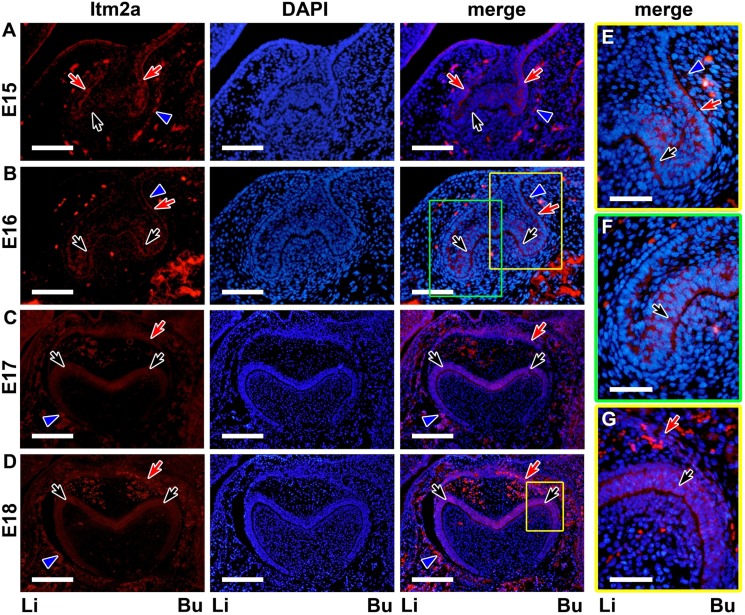
The Itm2a protein expression in the tooth germ on E15–E18. **A.** At the cap stage (E15), the inner enamel epithelium (black arrows) exhibited the expression of the Itm2a protein, but the signal was weak. A strong signal was observed in the outer enamel epithelium (red arrows) and dental sac (blue arrowheads). **B.** At the cap stage (E16), the inner enamel epithelium (black arrows) exhibited a strong signal for the Itm2a protein, as did the outer enamel epithelium (red arrows). **C & D.** At the early bell stage (E17–18), the Itm2a protein was detected in the outer enamel epithelium cells (red arrows), the inner enamel epithelium cells (black arrows) and dental sac (blue arrowheads). **E & F.** The yellow- and green-boxed areas in B are respectively shown at a higher magnification. **G.** The boxed areas in D are shown at a higher magnification. Li; lingual side, Bu; buccal side. Scale bars; 100 µm (A, B), 200 µm (C, D), 50 µm (E–G).

**Figure 7:**
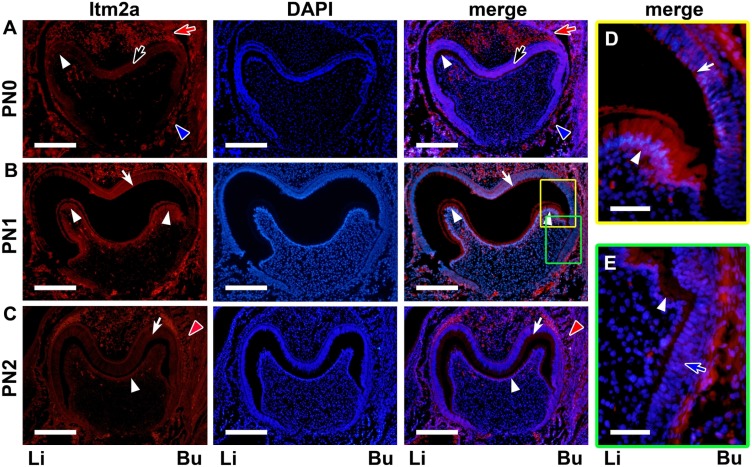
The Itm2a protein expression during tooth germ development on PN0–PN2. **A.** Itm2a protein expression was observed in the inner (black arrows) and outer (red arrows) enamel epithelial cells of neonatal mice. The protein signal was also detected in the dental sac (blue arrowheads). **B.** After starting matrix formation, a strong protein signal was observed in the ameloblasts (white arrows) and odontoblasts (white arrowheads). **C.** In addition to the ameloblasts (white arrows) and odontoblasts (white arrowheads), the osteoblasts (red arrowheads) of the bone tissue surrounding the tooth germ exhibited a strong protein signal. **D & E.** The yellow- and green-boxed areas in B are respectively shown at a higher magnification. Itm2a protein expression was observed in the preameloblasts (blue arrow). Li; lingual side, Bu; buccal side. Scale bars; 200 µm (A–C), 50µm (D, E).

**Figure 8:**
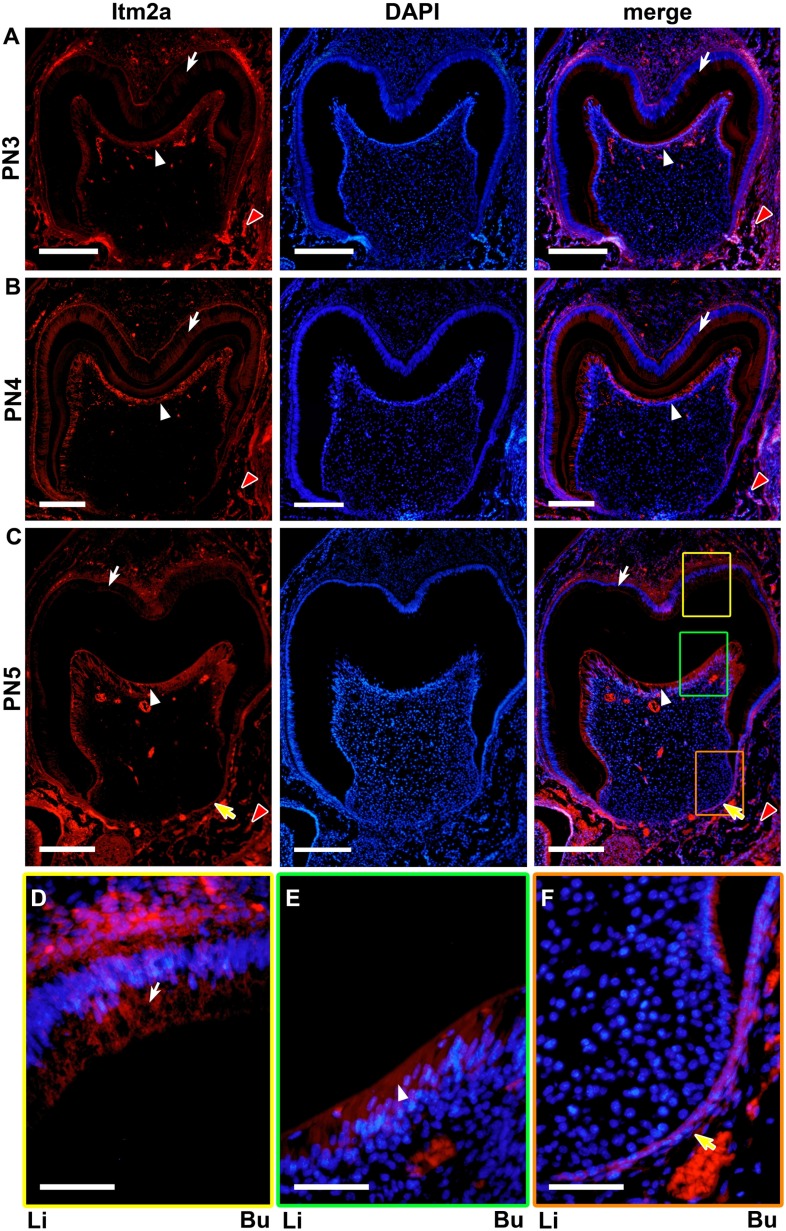
The Itm2a protein expression during tooth germ development on PN3-PN5. **A & B.** The ameloblasts (white arrows), odontoblasts (white arrowheads) and the osteoblasts (red arrowheads) of the bone tissue surrounding the tooth germ exhibited a strong protein signal. **C.** At the initiation stage of root formation, a strong Itm2a signal was observed in the odontoblasts (white arrowheads) and the osteoblasts (red arrowheads) of the bone tissue. The Itm2a signal was also detected in the ameloblasts (white arrows) and HERS (yellow arrows). **D–F.** The yellow-, green- and orange-boxed areas in C are respectively shown at a higher magnification. Li; lingual side, Bu; buccal side. Scale bars; 200 µm (A–C), 50 µm (D–F).

An immunofluorescent histochemical analysis (IHC) was also carried out using an anti-Itm2a antibody. On E10.5 and E12, Itm2a protein expression was not detected in the epithelial or mesenchymal cells corresponding to the predicted lower first molar region, nor was Itm2a mRNA (Fig. 5A, B, E). At the bud stage (E13–14), Itm2a protein expression was not observed in the epithelial cells of the tooth bud or in the mesenchymal cells condensed around the tooth bud (Fig. 5C, D, F). However, the protein expression of Itm2a in the subsequent stages was detected in the developing tooth germ, and demonstrated a similar expression pattern to that of the mRNA (Figs. 5–8). Some differences were identified, including a time lag, between the mRNA and protein expression, as indicated in the respective regions below.

Immunofluorescent signals for the Itm2a protein were observed in the enamel organ on E15 (Fig. 6A) and on the subsequent days, as was the signal for Itm2a mRNA. Although a signal for the mRNA was observed in the PEK on E15, a signal for the protein was undetectable in the PEK at that time. In the inner enamel epithelium, the signal for the protein became clearer on E16 (Fig. 6B, E, F) after the mRNA signal was detected. On E17-PN0 (Fig. 6C, D, G and Fig. 7A), a protein signal was detected in the inner enamel epithelium, but the intensity in the region near the cervical loop was weaker than that in the other regions at the presumptive cusp sites. A strong protein signal for Itm2a in the ameloblasts and preameloblasts on PN1 (Fig. 7B, D, E) and on the subsequent days demonstrated similar expression patterns to that of the mRNA signal observed on these days.

The outer enamel epithelium showed an immunofluorescent signal for protein expression on E15 (Fig. 6A) that was similar to that of the mRNA expression. On E16 (Fig. 6B, E, F), the signal intensity appeared to be increased on the buccal side. On E17 (Fig. 6C), the signal was clearly observed on both the buccal and lingual sides. The signal intensity became reduced after PN1, whereas the mRNA intensity decreased after PN0.

Immunofluorescent signals for Itm2a were also observed in the dental mesenchymal cells on E15 (Fig. 6A) and on the subsequent days. A strong protein expression was detected in the odontoblasts (Fig. 7B, D, E and Fig. 8), whereas mRNA expression was observed in the odontoblasts and preodontoblasts localized at the presumptive cusp sites of the dental pulp (Fig. 2). However, the protein signal was undetectable in the dental mesenchymal cells in the dental papilla, except for in the blood vessels (Figs. 6–8).

The dental sac exhibited strong protein expression on E15 (Fig. 6A), although its mRNA expression was weak on E15–18.

Therefore, both the mRNA and protein expression patterns were similar during odontogenesis. In addition, the expression of Itm2a was found in the developing skeletal muscles, the Meckel’s cartilage, the acini of exocrine glands such as sublingual glands, the brain, the retina, the lens and the osteoblasts. A strong signal was observed in the skeletal muscles, the acini of exocrine glands and the osteoblasts in this study.

### Subcellular localization of the Itm2a protein in the mouse dental epithelium

**Figure 9:**
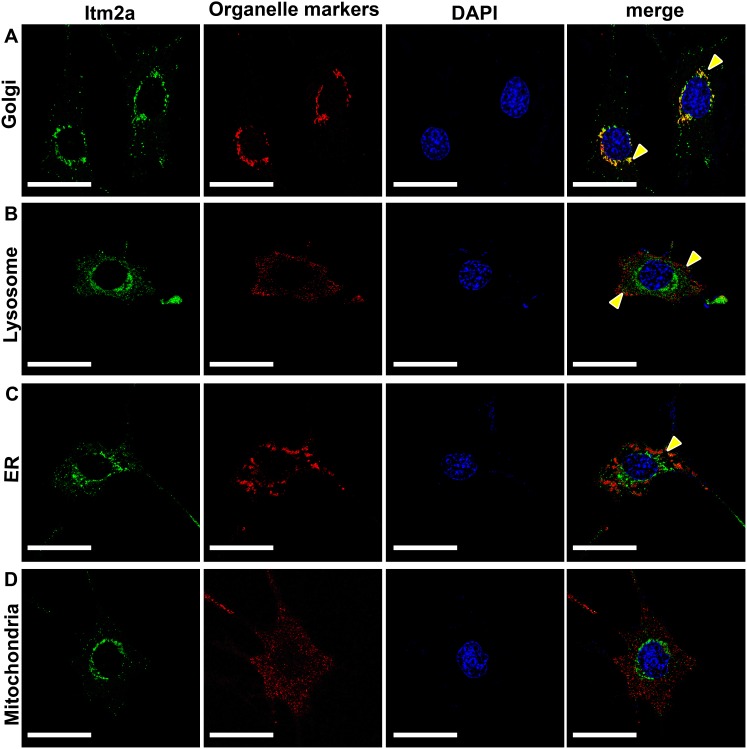
The subcellular localization of the Itm2a protein expression in cultured dental epithelial cells. The subcellular localization of the Itm2a protein in murine dental epithelial cells, mDE6 cells, which had been transfected with a pcDNA3.1(+)/Hygro vector encompassing Itm2a-EGFP cDNA (Itm2a-EGFP-mDE6 cells) was determined. The Itm2a in the Itm2a-EGFP-mDE6 cells was detected to have a punctate cytoplasmic distribution, which was shown as a green signal (left panels). The Itm2a-EGFP-mDE6 cells were stained with antibodies for cell organelle markers to indicate the cell organelles as follows: (**A**) Golgi apparatus, (**B**) lysosome, (**C**) endoplasmic reticulum (ER) and (**D**) mitochondria. These organelles were represented by red signals (second panels from the left). The nucleus was stained with DAPI (second panels from the right). The green signals indicating Itm2a, and red signals for each organelle and DAPI- counterstained nuclear images were merged, and are shown in the right panel. The yellow arrowheads indicate the colocalization of the organelle marker signal and Itm2a protein signal. Scale bars; 10 µm.

The subcellular localization of Itm2a was investigated in murine dental epithelial cells, mDE6 cells with stable Itm2a-EGFP expression (Fig. 9, left panels). The Itm2a-EGFP signal showed an apparent punctate pattern throughout the cytoplasm in the mDE6 cells. A large amount of the signal was detected in the perinuclear region. In some cells, the Itm2a-EGFP signal was localized to a part of the plasma membrane. Itm2a-EGFP was not detected in the nucleus. The subcellular localization of the Itm2a-EGFP protein demonstrated a similar expression pattern to that observed in the un-transfected mDE6 cells after immunocytochemistry using an antibody against Itm2a (data not shown), but the endogenous Itm2a signal was weak in the entire cytoplasm of the un-transfected mDE6 cells (Fig. S5).

We next performed immunocytochemistry using Itm2a-EGFP expressing mDE6 cells to investigate the Itm2a-EGFP localization in the cell organelles. Itm2a-EGFP displayed a frequent overlap with the Golgi apparatus marker, GM130 (Fig. 9A). A tiny part of the Itm2a-EGFP colocalized with the lysosome marker, LAMP2, near the plasma membrane (Fig. 9B). The partial colocalization of Itm2a-EGFP with the ER marker, PDI, was observed in this study (Fig. 9C). No colocalization of Itm2a-EGFP with the mitochondria marker, Mitofusion-2, was found in this study (Fig. 9D).

## Discussion

To clarify the role of Itm2a during odontogenesis, we investigated the temporal and spatial expression pattern of Itm2a in the developing lower first molar by *in situ* hybridization and immunohistochemical studies. We also examined the subcellular localization of Itm2a in the dental epithelial cells. On E10.5 and E12, the *in situ* signal for Itm2a was localized in the central region of the mandible, which corresponded to the lateral lingual swelling. On E14, the mRNA and protein were both detected in the muscular tissue of the tongue, but not in the epithelial cells of the tooth bud and the mesenchymal cells surrounding the tooth bud. These findings were identical to those of our previous study [5].

Meanwhile, on E15–16 and E17–18, the *in situ* signal for Itm2a mRNA was mainly observed in the inner enamel epithelium of the enamel organ (Fig. 1E–H), as was the signal for the Itm2a protein (Fig. 6A–D). Intriguingly, these strong signals were detected in ameloblasts and odontoblasts after the initiation of the matrix formation (Figs. 2, 3, 7B, C and 8A–C). At the initiation of the tooth root formation stage, the *in situ* signal intensity was reduced in the odontoblasts and ameloblasts in the presumptive cusp sites (Figs. 3D, E and 8C). Although there was a time lag between the mRNA and protein expression, the protein expression showed a similar pattern of expression as the mRNA. These findings regarding the Itm2a expression pattern suggest that Itm2a may play a role in the differentiation of dental epithelial cells and dental mesenchymal cells, as well as in myogenesis and chondrogenesis [24], [27]–[29]. The Itm2a expression may depend on the stage of development of enamel epithelial cells and odontoblasts. Although the precise functions of the BRICHOS domain remain unknown, three possible functions for this domain have been proposed: targeting proteins to a secretory pathway, an intramolecular chaperone-like function and assisting with a specialized intracellular protease processing system [21]. Thus, it is possible that Itm2a is also related to targeting the proteins associated with enamel and dentin matrices to a secretory pathway involved in the matrix formation.

The Itm2a-EGFP signal showed an apparent punctate pattern throughout the cytoplasm in the dental epithelial cells. The signal was mainly detected in the perinuclear region, and was not detected in the nucleus. In contrast, Itm2b (BRI2), another member of the gene family that includes Itm2a and Itm2c, was localized on the cell surface [33]. Therefore, different function(s) seem to be exhibited among members of the Itm2 family. In the immunocytochemical findings obtained using organelle markers, it was observed that the perinuclear-localized Itm2a-EGFP displayed a frequent overlap with the Golgi apparatus marker, GM130. Therefore, Itm2a might participate in the processing, packaging and/or transport of the macromolecules, such as proteins and lipids, in the Golgi apparatus. In addition, there was minimal or no overlap between the Itm2a-EGFP signals with the other organelle markers used in this study noted in the cytoplasm. Wang et al. [34] identified Itm2a as one of the protein partners of ameloblastin using the yeast two-hybrid assay. Together with the findings of previous reports [21], [34], these results suggest that Itm2a may contribute to enamel matrix formation via its interaction with ameloblastin in the secretory pathway in the ameloblasts. However, the weak endogenous Itm2a signals were localized throughout the entire cytoplasm, not in the perinuclear region, probably because the endogenous promoter used for Itm2a expression has relatively weak effects in the mDE6 cells. The co-localization of Itm2a and ameloblastin proteins was also undetectable in the mDE6 cells, probably because of the low amount of secreted ameloblastin present in the mDE6 cells.

In this study, we demonstrated the temporal expression patterns of Itm2a mRNA and protein in the developing tooth in mouse embryos and neonates, and thus showed the close association of this gene with odontogenesis. Based on the expression pattern in the developing tooth germ, Itm2a is considered to be related to matrix formation in the late bell stage. Itm2a also appears to participate in cell differentiation. In terms of its subcellular localization, Itm2a showed a punctate pattern in the cytoplasm of the dental epithelial cells whereas Itm2b protein, which is another member of the Itm2 family, was located in the plasma membrane [33]. Thus, because Itm2a possesses diverse functional properties during odontogenesis, further investigations, including in vivo studies, will be necessary to understand the biological function of Itm2a and the significance of the interaction between Itm2a and ameloblastin during odontogenesis.

## Supporting Information

Figure S1 **The membrane hybridization of the** ***in situ*** **probes, and the** ***in situ*** **expression of Itm2a in the tooth germ detected with a DIG-labeled sense probe.** **A.** An antisense probe for Itm2a mRNA showed dose-dependent hybridization with Itm2a sense RNA. No hybridization of the DIG-labeled Itm2a anti-sense probe was observed with Itm2a antisense RNA. B. The binding activity of the Itm2a antisense probe to the Itm2a sense probe was inhibited by adding excess unlabeled sense or antisense probe to the reaction mixture. **C-E.** No *in situ* signal was found in the tissue sections treated with the DIG-labeled Itm2a sense probe. (**C**) E15, (**D**) E17 and (**E**) PN3. Scale bars; 100 µm (C-E).
(TIF)

Figure S2 **An immunofluorescent section of paraffin-embedded mouse liver used as a control.**
(TIF)

Figure S3 **A fluorescent image of cultured dental epithelial cells transfected with the pEGFP-N1 vector.**
(TIF)

Figure S4 **An immunofluorescent image of Itm2a-EGFP-mDE6 cells incubated with an antibody against Itm2a.** A. The Itm2a in the Itm2a-EGFP-mDE6 cells had a punctate cytoplasmic distribution, which was shown as a green signal. **B.** The Itm2a-EGFP-mDE6 cells were stained with an antibody against Itm2a. The Itm2a protein was represented by red signals. **C.** The nucleus was stained with DAPI. **D.** These images were merged, and are shown in panel D. The yellow signals indicate the colocalization of the Itm2a-EGFP and signals detected by the antibody. Scale bars; 10 µm.
(TIF)

Figure S5 **An immunofluorescent image of the endogenous Itm2a protein expression in cultured dental epithelial cells.** The endogenous Itm2a in some un-treated mDE6 cells was detected to have a punctate cytoplasmic distribution, which was shown by weak red signals. The nucleus was stained with DAPI. The red signals indicating Itm2a and DAPI-counterstained nuclear images were merged. Scale bars; 10 µm.
(TIF)
