# Supplementary figures and images for: Itm2a Expression in the Developing Mouse First Lower Molar, and the Subcellular Localization of Itm2a in Mouse Dental Epithelial Cells
Source: PLoS One. 2014 Jul 31;9(7):e103928. doi: 10.1371/journal.pone.0103928 (PMC4117645; doi:10.1371/journal.pone.0103928)

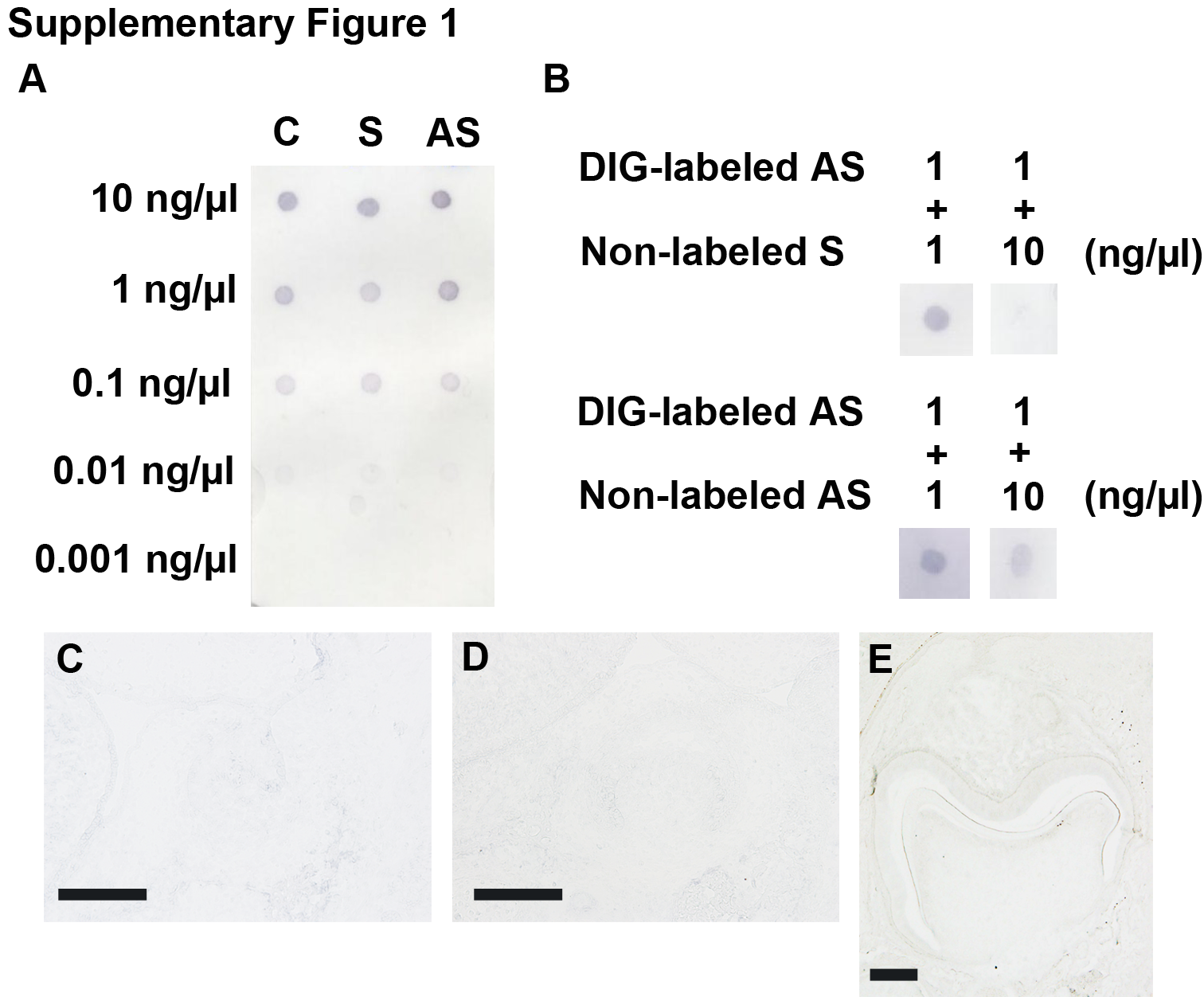

Supplement: Figure S1 — The membrane hybridization of the in situ probes, and the in situ expression of Itm2a in the tooth germ detected with a DIG-labeled sense probe. A. An antisense probe for Itm2a mRNA showed dose-dependent hybridization with Itm2a sense RNA. No hybridization of the DIG-labeled Itm2a anti-sense probe was observed with Itm2a antisense RNA. B. The binding activity of the Itm2a antisense probe to the Itm2a sense probe was inhibited by adding excess unlabeled sense or antisense probe to the reaction mixture. C-E. No in situ signal was found in the tissue sections treated with the DIG-labeled Itm2a sense probe. (C) E15, (D) E17 and (E) PN3. Scale bars; 100 µm (C-E). (TIF) [file pone.0103928.s001.tif]

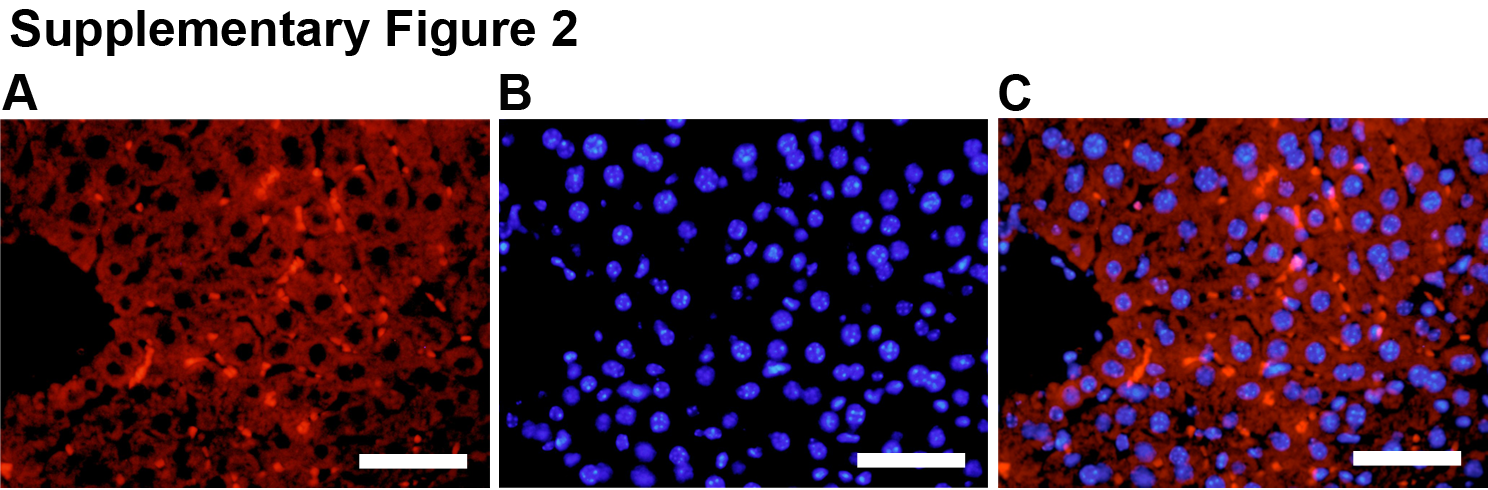

Supplement: Figure S2 — An immunofluorescent section of paraffin-embedded mouse liver used as a control. (TIF) [file pone.0103928.s002.tif]

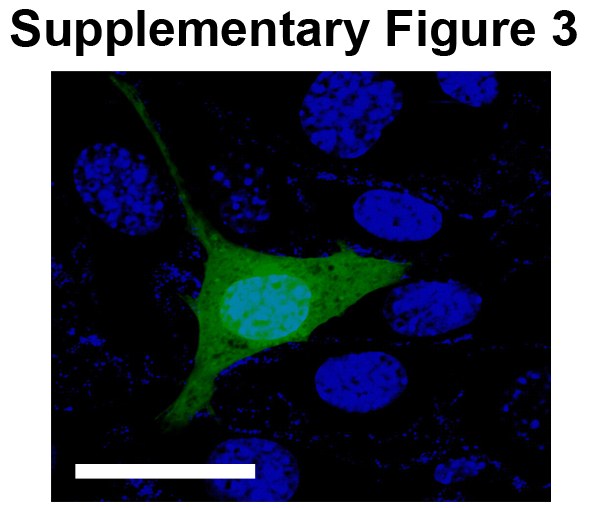

Supplement: Figure S3 — A fluorescent image of cultured dental epithelial cells transfected with the pEGFP-N1 vector. (TIF) [file pone.0103928.s003.tif]

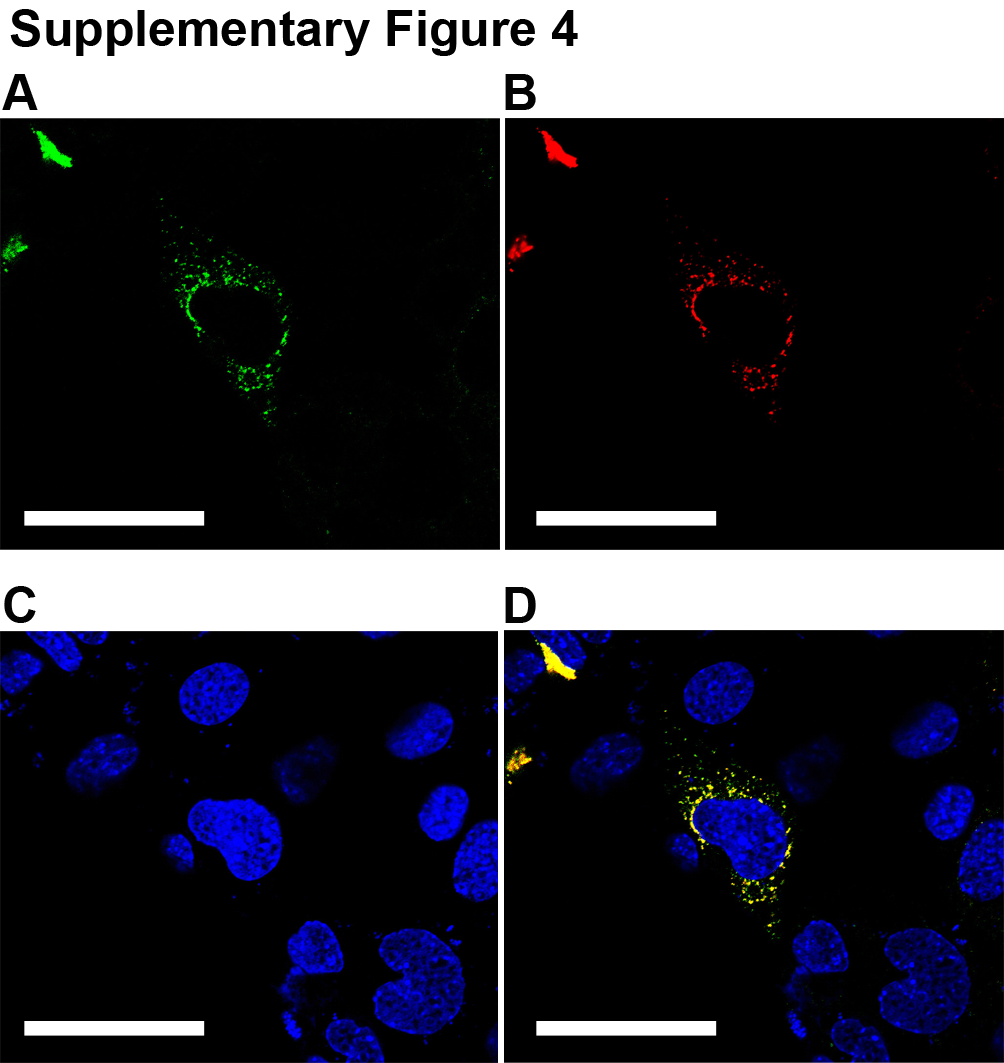

Supplement: Figure S4 — An immunofluorescent image of Itm2a-EGFP-mDE6 cells incubated with an antibody against Itm2a. A. The Itm2a in the Itm2a-EGFP-mDE6 cells had a punctate cytoplasmic distribution, which was shown as a green signal. B. The Itm2a-EGFP-mDE6 cells were stained with an antibody against Itm2a. The Itm2a protein was represented by red signals. C. The nucleus was stained with DAPI. D. These images were merged, and are shown in panel D. The yellow signals indicate the colocalization of the Itm2a-EGFP and signals detected by the antibody. Scale bars; 10 µm. (TIF) [file pone.0103928.s004.tif]

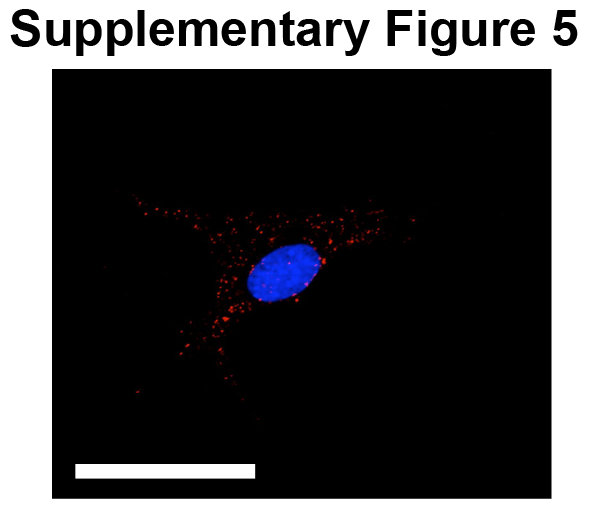

Supplement: Figure S5 — An immunofluorescent image of the endogenous Itm2a protein expression in cultured dental epithelial cells. The endogenous Itm2a in some un-treated mDE6 cells was detected to have a punctate cytoplasmic distribution, which was shown by weak red signals. The nucleus was stained with DAPI. The red signals indicating Itm2a and DAPI-counterstained nuclear images were merged. Scale bars; 10 µm. (TIF) [file pone.0103928.s005.tif]
